# Super-Enhancers in Placental Development and Diseases

**DOI:** 10.3390/jdb13020011

**Published:** 2025-04-09

**Authors:** Gracy X. Rosario, Samuel Brown, Subhradip Karmakar, Mohammad A. Karim Rumi, Nihar R. Nayak

**Affiliations:** 1 Department of Obstetrics and Gynecology, University of Missouri-Kansas City, Kansas City, MO 64108, USA; srbnc5@health.missouri.edu (S.B.); nnayak@umkc.edu (N.R.N.); 2 Department of Biochemistry, All India Institute of Medical Sciences, New Delhi 110029, India; subhradip.k@aiims.edu; 3 Department of Pathology and Laboratory Medicine, University of Kansas Medical Center, Kansas City, KS 66160, USA; mrumi@kumc.edu

**Keywords:** super-enhancers, transcriptional regulators, trophoblast stem cells, placental development, placental diseases

## Abstract

The proliferation of trophoblast stem (TS) cells and their differentiation into multiple lineages are pivotal for placental development and functions. Various transcription factors (TFs), such as CDX2, EOMES, GATA3, TFAP2C, and TEAD4, along with their binding sites and cis-regulatory elements, have been studied for their roles in trophoblast cells. While previous studies have primarily focused on individual enhancer regions in trophoblast development and differentiation, recent attention has shifted towards investigating the role of super-enhancers (SEs) in different trophoblast cell lineages. SEs are clusters of regulatory elements enriched with transcriptional regulators, forming complex gene regulatory networks via differential binding patterns and the synchronized stimulation of multiple target genes. Although the exact role of SEs remains unclear, they are commonly found near master regulator genes for specific cell types and are implicated in the transcriptional regulation of tissue-specific stem cells and lineage determination. Additionally, super-enhancers play a crucial role in regulating cellular growth and differentiation in both normal development and disease pathologies. This review summarizes recent advances on SEs’ role in placental development and the pathophysiology of placental diseases, emphasizing the potential for identifying SE-driven networks in the placenta to provide valuable insights for developing therapeutic strategies to address placental dysfunctions.

## 1. Introduction

The placenta is a highly specialized organ crucial for fetal development, formed through the proliferation and differentiation of trophoblast stem (TS) cells [1]. The proliferation of TS cells and their differentiation into multiple lineages are pivotal for placental development and function [2,3]. The placenta’s development is governed by intricate gene regulatory mechanisms, ensuring the proper formation of structures essential for maternal–fetal interaction. As TS cells differentiate into cytotrophoblasts, these cells give rise to extravillous trophoblasts that invade the maternal decidua and remodel the spiral arteries, establishing vital maternal–fetal blood flow [4,5,6,7]. The syncytiotrophoblast, which is formed via the fusion of cytotrophoblasts, is responsible for exchanging gases and nutrients between the mother and fetus. Aberrant trophoblast differentiation, defective invasion, and syncytiotrophoblast dysfunction are associated with a variety of pregnancy complications, including preeclampsia (PE), fetal growth restriction, and gestational trophoblastic disease (GTD), highlighting the critical role of precise regulatory pathways in placental health and function [8,9,10,11,12]. While various gene regulation mechanisms have been studied for their roles in trophoblast cells, much about their precise regulatory functions remains poorly understood.

Cell proliferation and differentiation are dependent on lineage-specific gene expression. Moreover, a well-regulated gene expression pattern is the cornerstone for maintaining unique cell identity. Such genetic homeostasis at the cellular level is accomplished through coordination at multiple levels, including the epigenetic regulation of chromatin architecture, binding of transcriptional regulators to cis-regulatory DNA elements, and interaction of the RNA polymerase components [9]. Transcription factors (TFs), including caudal type homeobox 2 (CDX2), eomesodermin (EOMES), transcription factor AP-2 gamma (TCFAP2C), GATA binding protein 3 GATA3, ETS proto-oncogene 2 transcription factor (ETS2), E74-like ETS transcription factor 5 (ELF5), estrogen-related receptor beta (ESRRB), SRY-box transcription factor 2 (SOX2), inhibitor of DNA binding 1 (ID1), ID2, TEA domain transcription factor 4 (TEAD4), SATB homeobox 1 (SATB1), and SATB2, have been identified as key regulators of trophoblast lineage determination [13,14,15]. However, although some controversies remain, the emerging literature suggests that distinct species-specific transcription factors (TFs) regulate TS cell differentiation and function in humans and mice [16,17]. While TFs such as CDX2 and EOMES may play critical roles in human iTP cell generation, a comparative study of mouse and human placentae across gestation identified species-specific regulators of placental development [16]. Notably, VGLL1, which is co-expressed with TEAD4, was identified as a human-specific marker of proliferative cytotrophoblasts, whereas CDX2 and ELF5 were expressed in early trophoblast subpopulations in both species, but EOMES was not [17]. The regulatory DNA elements to which the TFs bind, are also decisive for regulating trophoblast functions [13,14,18,19]. Studies have shown that sequence variation within enhancer regions can impact trophoblast development [13,14,18,19].

While most previous studies have emphasized the roles of individual transcriptional regulators in trophoblast development or differentiation, recent studies have highlighted the cluster of enhancers enriched in TF-binding, vital for trophoblast lineage determination and placental development. Such clusters of enhancers are known as super-enhancers (SEs). This review article has summarized the role of SEs in TS cell proliferation, lineage-specific trophoblast differentiation, placental development, and placental diseases.

## 2. Super-Enhancers

**Figure 1:**
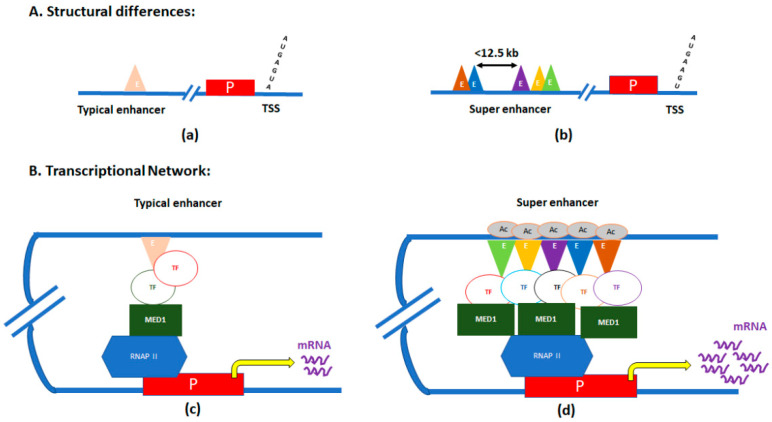
Graphic illustration of differences between typical enhancers and super-enhancers (SEs). (**A**) Structural differences between typical enhancers (**a**) and SEs (**b**). (**B**) Transcriptional loops/networks of typical enhancers (**c**) and SEs (**d**). E, enhancer regions; P, promoter of the gene; TSS, transcription start site; TF, transcription factor; MED, mediator subunit 1; RNAP II, RNA polymerase II; and Ac, acetylation.

SEs are clusters of enhancers located in close proximity, and with significantly elevated binding of the transcription coactivator Mediator complex [20,21]. SEs were first identified in ChIP-Seq experiments of three master regulators of pluripotency—POU class 5 homeobox 1 (OCT4), SOX2, and nanog homeobox (NANOG) in mouse embryonic stem (ES) cells [20,22]. Those genes were also associated with other pluripotency factors like SOX2, Pim-1 proto-oncogene, serine/threonine kinase (PIM1), and fibroblast growth factor receptor 1 (FGFR1), which are evolutionarily conserved in mammals to maintain pluripotency in stem cells [23]. In a single SE, numerous enhancers in proximity (<12.5 kb) are stitched together (Figure 1) [24].

The mediator subunit MED1’s binding levels are significantly elevated within these ‘stitched’ genomic regions. The mediator binds with the chromatin-associated protein cohesin, forming the mediator–cohesin complex, thereby facilitating formation of DNA loops to physically connect enhancers with core promoters to activate transcription [25]. As the mediator–cohesin complex occupies different promoters in different cell types, specific cell-type DNA loops are formed. Given this fact, along with the characteristic enrichment of Mediator and cohesin at SEs, SEs may act as directors of cellular identity through cell-type-specific reorganization of 3D chromatin architecture [3,25]. Thus, SEs can act as ‘switches’ that control the expression of specific regulatory genes determining cell fate.

In addition to MED1, multiple pro-transcriptional regulators, which are hallmarks of active transcription, are enriched at SEs. For example, the enriched binding of RNA polymerase II and the histone acetyltransferases p300 and creb-binding protein (CBP), cohesion structural maintenance of chromosomes (SMC) complexes, and chromatin remodeling factors, have all been found within SE regions [20,26]. Furthermore, high levels of enhancer-specific H3 lysine 27 acetylation marks have also been associated with SEs [3,27].

## 3. Placental Development

**Figure 2:**
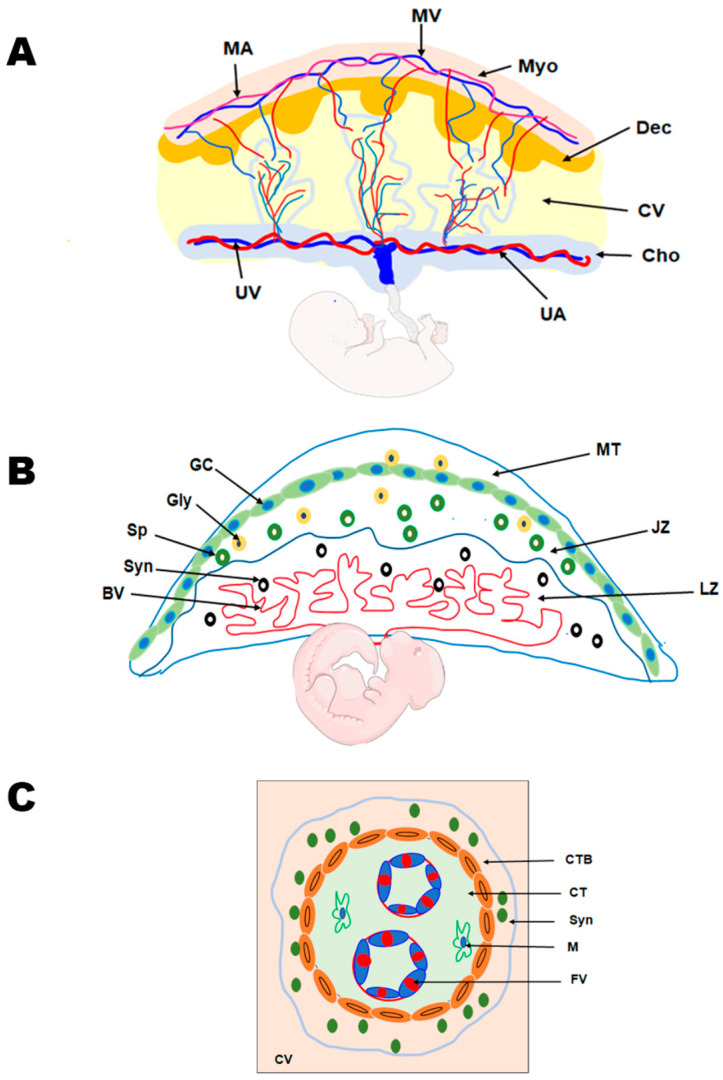
Comparative anatomy of human and mouse placenta. (**A**) Schematic representation of the human placenta illustrating the maternal and fetal surfaces, which are attached by the maternal decidua and the associated vasculature. MA, maternal artery; MV, maternal vein; Myo, myometrium; Dec, decidua; CV, chorionic villus; Cho, Chorion; UA, umbilical artery; and UV, umbilical vein. (**B**) Schematic representation of the rodent placenta showing the labyrinth (LZ) and junctional zones (JZ) that are overlaid by the mesometrial triangle (MT). The labyrinth zone is rich in maternal and fetal blood vessels (BV) and syncytiotrophoblast (Syn) cells. The junctional zone, overlying the labyrinth zone, mainly contains the spongiotrophoblast (Sp), glycogen cells (GC), and the giant cells (GC). (**C**) Schematic representation of the chorionic villus in the human placenta demonstrating the cytotrophoblast (CTB), syncytiotrophoblast (Syn) cells, and the connective tissue constituting of macrophages (M) and the fetal vessels (FVs).

Trophoblasts, specialized epithelial cells in the placenta, are the primary cell type responsible for its development. They play a crucial role in embryo implantation and establishing the essential connection between the embryo and the maternal decidua, facilitating the formation of the maternal–fetal interface. As the precursor for all trophoblast cell types in the placenta, the blastocyst’s multipotent trophectoderm ensures proper trophoblast lineage determination. In humans, the trophectoderm is formed approximately 4–5 days post fertilization and consists of TS cells. The TS cells will differentiate into cytotrophoblasts, which undergo several rounds of proliferation, breaking through the primary syncytium to form the primary villi. These villous cytotrophoblasts will continue to proliferate and undergo fusion to generate the secondary villi [18,19]. The multinucleated syncytiotrophoblast forms when villous cytotrophoblasts proliferate asymmetrically. This asymmetric proliferation is followed by differentiation and fusion with the syncytium, forming tertiary villi comprising fetal blood vessels, thereby establishing the maternal–fetal blood and nutrient exchange (Figure 2) [28]. The cytotrophoblasts at the anchoring villi that attach the placenta to the uterine wall form the cell column trophoblasts. These cell column trophoblasts will differentiate into the extravillous trophoblast cells that are involved in vascular remodeling. The extravillous trophoblasts further differentiate into two types of cells—endovascular extravillous trophoblasts that remodel the maternal spiral arteries and the interstitial extravillous trophoblasts that remodel the maternal decidual stroma [28,29,30].

Information on human placental development in vivo is limited, as studies have primarily been conducted on term placenta, primary placental cultures, choriocarcinoma, and immortalized cell lines due to ethical constraints. Hence, placental development has been increasingly studied using rodent models. Both humans and rodents display hemochorial placentation, in which the trophoblasts directly interact with maternal blood; however, the placental structure and types of trophoblast cells vary between the two species. The ease of genetic manipulation, shorter pregnancy, genetic uniformity, easy availability, and presence of human orthologous genes in mice make mouse models attractive for studying placental pathophysiology. The trophectoderm (TE) of the mouse blastocyst is classified as mural TE or polar TE. The polar TE, which lies in contact with the inner cell mass (ICM), undergoes rapid proliferation to generate the extraembryonic ectoderm and ectoplacental cone. Conversely, the mural TE, lying away from the ICM, differentiates into multinucleated trophoblast giant cells (TGCs) [10,30,31,32]. As pregnancy proceeds, the ectoplacental cone gives rise to trophoblast cells of the labyrinth and junctional zones. The labyrinth zone has three types of trophoblast cells—multinucleated syncytiotrophoblast I and II and mononucleated sinusoidal giant cells. The junctional zone, which lies above the labyrinth zone, consists of spongiotrophoblast cells, glycogen cells, and TGCs [28,33,34] (Figure 2). The glycogen cells that invade the stroma are the interstitial invasive trophoblasts, and those that invade the blood vessels are known as endovascular trophoblasts [32,35]. It is also reported that glycogen cells can invade the maternal decidua and the overlying maternal metrial gland to a lower extent [36].

## 4. Role of Super-Enhancers in Placental Development

### 4.1. Super-Enhancers in Trophectoderm Development and Trophoblast Differentiation

The known association of SEs with master pluripotent factors has prompted recent investigations on studying SEs in trophectoderm differentiation. Still, the idea that SEs may act as the drivers of trophectoderm differentiation is a relatively new concept. Although several TF regulators of trophoblast lineage specification have been identified historically, the role of SEs in driving large-scale identity-dependent gene expression has become more evident following advances in genome mapping.

Currently, it is hypothesized that the epigenetic regulation of the trophectoderm genome may be a foundation for proper fetal development and healthy pregnancy. In mice, one identified SE-associated transcription factor is the caudal homeobox transcription factor, CDX2. RNA-Seq and ChIP-In mice seq analyses revealed that CDX2 is associated with SEs in embryonic tissues and acts as a regulator of trophoblast self-renewal and early lineage specification [1]. In addition, a time-dependent expression pattern of CDX2 within the trophectoderm was observed, where high levels of the TF were gradually downregulated as TS cells underwent differentiation [1,37]. CDX2 expression is weak within the mouse placenta, restricted to trophectoderm precursor cells, leading to the postulation that the SE-associated TF is primarily involved in trophoblast self-renewal and early lineage specification [1,37]. Concurrent with the tight spatial and temporal expression patterns of CDX2, Strumpf et al. reported that proper expression of SE-associated CDX2 is required for the segregation of the ICM from the remainder of the blastocysts and subsequent functional trophectoderm development [37]. In humans, CDX2 is expressed by 5-day post-fertilization blastocysts, although its trophectoderm expression lags by 2 days compared to the mouse [38]. CDX2 is weakly expressed by cytotrophoblast cells and human-induced trophoblast progenitor cells [16,17,39,40]. However, its role seems to be less critical in humans than in mice, as human trophoblast development and differentiation depend more on other factors.

The determination of trophoblast lineage is a highly complex process, dependent on a complex network of TFs. Specifically, mapping such SE-associated TFs revealed “highly intertwined transcriptional regulatory circuitry”, but distinct classes of expression patterns [1]. Within this complex intra-regulatory network, the evidence suggests that SE-associated-TFs regulate each other collaboratively, without any clear hierarchal structure between TFs. For example, in addition to CDX2’s role in driving trophectoderm differentiation, CDX2 is also known to regulate the activity of another SE-associated TF, OCT4, to ensure proper blastocyst differentiation in rodents. While CDX2 and OCT4 have been deemed ‘prime movers’ of the first cell-specific lineages, these TFs have separate and occasionally mutually exclusive effects [41]. Specifically, CDX2 plays a role in ICM segregation and trophectoderm differentiation, while OCT4 is an ICM-specific TF required to maintain ICM fate and embryonic stem cell pluripotency [37]. The roles of CDX2 and OCT4 in a complex and collaborative intra-regulatory network may instead imply a separate hierarchical structure. While current data suggest a lack of stable hierarchy among those TFs that bind and activate SEs, transitory hierarchal structures may exist secondarily to competing TF expression levels. It has been hypothesized that proper blastomere differentiation into trophectoderms or ICM may be predicated upon an epigenetic tug-of-war relationship between CDX2 and OCT4 levels and the SE-associated genes they each activate. Current evidence suggests that the ratio of CDX2 to OCT4 expression determines trophectoderm fate. Specifically, Niwa et al.’s combined qPCR and stem cell luciferase reporter experiments demonstrated that OCT4 expression is conserved and consistent across all blastomeres of early morulae [42]. As the outer layer of cells differentiates into trophectoderms during blastocyst formation, the expression of OCT4 is suppressed while CDX2 increases [37]. Before blastocyst formation, a high OCT4–CDX2 ratio (OCT4 > CDX2) was shown to promote the maintenance of the ICM, whereas a low OCT4–CDX2 ratio (CDX2 > OCT4) stimulated differentiation into trophectoderms [37,42]. However, these intricate interactions between CDX2 and OCT4 have not been fully explored in human trophoblast cells and should be extrapolated to human studies with caution, as the suppression of OCT4 by CDX2, observed in mouse embryos and embryonic stem cells, was not observed in a study using human trophoblast cells derived from ES and iPS cells [43].

Recent evidence suggests that the expression levels of master TFs, CDX2, and OCT4 are determined by two factors: an interconnected autoregulatory feedback loop and SEs [22]. More specifically, it has previously been shown that master TFs (including OCT4, SOX2, and NANOG) form an interconnected autoregulatory loop in murine embryonic stem cells, whereby all three TFs bind as a group to the promoters of each of their genes to regulate transcription [22]. Furthermore, using a combination of ChIP-Seq and OCT4 luciferase reporters, Whyte et al. reported that many of the enhancers occupied by OCT4 were associated with the genes OCT4 and CDX2 themselves [22]. Given SEs’ role in driving higher-level transcription and associating with these master TFs, SEs may function as types of master regulators responsible for activating specific TF expression profiles and maintaining appropriate stoichiometric ratios of CDX2 and OCT4.

Recently, SEs have been increasingly implicated in cell differentiation events linked to generating various trophoblast lineages from TS cells. The SE-bound TFs that are associated with mouse trophoblast lineages have been categorized into classes 1–4 based on hierarchical clustering analysis [1]. Within these classes, the class 2 TFs are associated with the maintenance of TSC self-renewal and trigger early trophectoderm lineage specification, while class 3 TFs are highly expressed in the placenta and involved in trophoblast differentiation pathways [1]. The interactions between these different classes of SE-associated TFs, based on their stoichiometric ratios in the cell determine whether the TSC maintains its identity or differentiates into other cell lineages. For example, ELF5, a class 2 TF, aids in TSC self-renewal and triggers trophoblast differentiation networks via interactions with EOMES and TFAP2C [44,45,46,47]. The presence of equal stoichiometric quantities of EOMES, ELF5, and TFAP2C proteins in TS cells results in the binding of ELF5 to EOMES. The ELF5–EOMES interaction triggers the recruitment of TFAP2C protein to triple occupancy sites located in genes for TSC identity. Conversely, when levels of ELF5 and TFAP2C exceed those of EOMES, ELF5 directly interacts with TFAP2C. The ELF5–TFAP2C interaction triggers the recruitment of TFAP2C to double- and single-occupancy TFAP2C motifs in differentiation-specific genes for the TS cells to undergo differentiation [44].

Accurate trophectoderm development and trophoblast lineage differentiation may be directed by higher-level SEs that maintain a balance of isolated temporospatial TF expression within a complex regulatory network. The efficient CDX2–OCT4 balance, regulated by SEs, may control mouse ICM maintenance and/or trophectoderm cell fate determination. Conversely, trophoblast cell differentiation may be determined by specific SE-mediated transcription loops, wherein the TFs bind in a coordinated manner to initiate downstream effects. Further studies on how such transcription loops are intricately triggered by SE during placentation could provide valuable clues on the mechanisms involved in trophectoderm/trophoblast differentiation.

### 4.2. Super-Enhancers in Trophoblast Invasion

The invasion of trophoblasts into the maternal tissue during pregnancy promotes blood flow to the placenta via spiral artery remodeling. Several gene networks involved in trophoblast invasion for spiral artery remodeling have been identified by studies on rodents [48,49]. These gene expression networks coordinate the expression of cell adhesion molecules, growth factors, extracellular matrix proteins, and TFs. Recent studies have mapped epigenetic modifiers (such as DNA methylation and histone acetylation) and generated subsequent knockout models to assess changes in the invasion phenotype to better understand the underlying epigenetic landscape required for placental development and trophoblast invasion [26,50]. Thus, epigenetic regulation may act as a foundation for proper trophoblast invasion [51].

SEs have been increasingly implicated as drivers of the expression levels necessary for trophoblast invasion (Table 1). As human TS cells gain an invasive phenotype, increased enhancer-driven gene regulatory networks are detected in these extravillous trophoblast cells [52]. ATAC-Seq identified 1283 SEs in regulatory regions specific to extravillous trophoblast cells [52]. In Tuteja et al.’s ChIP-Seq of trophoblast invasion gene enhancers and accompanying trans-binding TFs, three TFs were identified as most likely to bind migration enhancers: fos proto-oncogene, AP-1 transcription factor subunit 1 (FOSL1), ETS2, and TFAP2C [13,53,54,55]. Identifying FOSL1 was especially notable, as Lee et al. found the TF to be SE-associated, undergoing gradual upregulation throughout trophoblast differentiation [1,13,56,57]. Furthermore, the TF is known to dimerize with jun proto-oncogene (JUN) proteins to form the AP-1 complex, a transcriptional regulator of cell survival, proliferation, and migration [56,57]. A downstream target of AP-1 is matrix metalloproteinase 9 (MMP9) of the matrix metallopeptidase family of proteases. Involved in cell invasion and extracellular matrix remodeling, MMP9is preferentially expressed in endometrial stromal cells and natural killer cells (NK) cells [13,58,59,60]. MMP9 is also known to be constitutively expressed in placental bed trophoblasts, where it is upregulated throughout pregnancy. There is no concrete evidence of SE regulation of MMP9 in trophoblasts, although more recent findings suggest MMP9 to be associated with some SEs. For example, Chandra et al.’s recent transcriptomic analysis revealed SEs’ regulation of MMP9 within neutrophils [58]. Furthermore, Tuteja et al.’s analysis of enhancers associated with trophoblast invasion revealed the presence of an E7.5, a timepoint-specific, potent enhancer of MMP9 [13,14]. MMP9 has been implicated in several disease states, as weak or absent expression has been found in PE, while the knockdown of the gene is lethal and associated with abnormal implantation [1,13,59,61].Another relevant TF that may be relevant to placentation is the TF TFAP2C. Initially, Lee et al. identified the association of TFAP2C with SEs. It was further confirmed by Tuteja et al. that TFAP2C acts on a SE during trophoblast migration [1,13]. This TF has been previously implicated in trophectoderm lineage determination and placental development, while RNA-Seq revealed changes in TFAP2C expression during trophoblast invasion [15]. Using ChIP-Seq analyses, Tuteja et al. reported TFAP2C as one of a few genes whose enhancer enrichment changed during the height of trophoblast invasion at E7.5 [13]. Furthermore, TFAP2C expression was observed in invasive extravillous trophoblasts, while TFAP2C knockdown resulted in embryo lethality secondary to a placental defect [62]. TFAP2C aids in the differentiation of extravillous trophoblast cells and the conversion of fibroblast cells into trophoblast cells [52]. As such, TFAP2C exemplifies the evolving notion of SE involvement in regulating trophoblast invasion.

In summary, SE-targeted TFs have been identified as drivers of trophoblast invasion, although underlying regulatory mechanisms of invasion appear highly complex and are presently poorly understood. Continued inquiry into the identities and impact of both trans-acting TFs and cis-acting SEs on gene expression will be needed to improve our understanding of the specific roles of SEs in directing trophoblast invasion.

### 4.3. Super-Enhancers in Placental Vascular Remodeling

Vascular remodeling events during trophoblast invasion are tightly coordinated cellular processes, converting maternal spiral arteries into “low resistance, high capacitance vessels”, for increased blood flow to the fetus [63,64,65,66]. The disruption of these vascular remodeling events leads to pregnancy-related complications such as PE [67]. With the advent of genome sequencing and mapping, the role of SEs has become increasingly apparent in vessel formation and endothelial homeostasis (Table 1). One such regulator of vascular remodeling is the SE-associated TF, ERG. ERG is a member of the ETS family, downstream targets of the RAS/ERK pathway [21]. ERG is primarily known to maintain vascular homeostasis and promote vascular development and angiogenesis through the regulation of several genes, including cadherin 5 (CDH5), angiopoietin (ANGP2), delta-like canonical notch ligand 4 (DLL4), claudin 5 (CLDN5), and Von Willebrand factor (VWF) [56,66,68]. In 2018, Kalna et al. revealed the role of ERG in key endothelial gene SEs by binding enriched DNA-enhancer regions and binding and recruiting histone acetyltransferases in HUVECs [56]. Furthermore, Lee et al.’s mapping of trophoblast regulatory networks revealed the increased expression of ERG in the placenta, with gradual upregulation throughout trophoblast differentiation [1]. Consistent with these findings, several studies have shown that ERG knockout leads to severe vascular dysfunction and subsequent embryo death [69,70]. Furthermore, environmental factors, such as hypoxia and reactive oxygen species, can upregulate ERG expression, as can endogenous growth factors [66]. Notably, the proangiogenic factor VEGF has been shown to upregulate ERG expression in human umbilical vein endothelial cells (HUVECs), inducing increased ETS promotor binding, expression of ANGP2, and subsequent vessel destabilization necessary for angiogenesis [66,71].

The GATA family of TFs has also been implicated in the SE-mediated regulation of vascular development. The GATA family comprises six isoforms and functions as zinc-coordinated regulatory proteins that bind to consensus DNA regions to regulate gene transcription [51,72]. Although GATA family members are perhaps best known as key regulators of trophectoderm lineage determination, GATA-2 and GATA-3 have been shown to help epigenetically coordinate vascular development [73,74,75,76]. Known to be associated with SEs and having multiple histone acetyltransferase target sites, GATA-3 regulates the expression of the peptide hormone PLF, along with GATA-2 [51,77]. A key positive and negative angiogenic regulator, PLF expression peaks at mid-gestation and stimulates endothelial cell migration, neovascularization, and angiogenesis within murine TGCs [1,51]. Furthermore, SEs may also play a significant role in maintaining the overarching epigenetic coordination for proper placental vascular development. Such studies are valuable for understanding placental diseases hampering fetal development due to aberrant nutrient and gas exchanges caused by defective placental vascular remodeling.

**Table 1:** Super-enhancer genes involved in placental development. (**A**) trophectoderm development and trophoblast differentiation; (**B**) trophoblast invasion; and (**C**) vascular remodeling.

Trophoblast-Specific TFs	SE Association	Trophoblast Placental Sub-Cell Types	Species	Functions	References
CDX2	Intra-regulatory network with OCT4 TF.	Trophoblast progenitor cells	Mouse	Trophoblast self-renewal and early lineage specification. Inner cell mass (ICM) segregation and trophectoderm differentiation.	[1,17,37,39,40,41,43]
ELF5	Recruitment of TFAP2C protein to double/single- (Interaction with TFAP2C) or triple-occupancy sites (Interaction with EOMES) in genes for TS cell identity or differentiation.	TS cells, Villous cytotrophoblast cells	Human and mouse	TSC self-renewal and trophoblast differentiation.	[44]
EOMES	Interacts with ELF5 to recruit TFAP2C to triple-occupancy sites in TS cell identity genes.	TS cells	Mouse and human	TS cell identity.	[44,45,46,47]
TFAP2C (human) or TCFAP2c (mouse)	Interacts with TFAP2C to recruit TFAP2C to double- or single-occupancy sites in differentiation genes in TS cells.	TS cells, Invasive trophoblast cells	Mouse and human	Involved in trophectoderm lineage determination, placental development, and trophoblast differentiation.	[13,44,45,46,47]
**Trophoblast-Specific TFs**	**SE Association**	**Trophoblast/Placental Sub-Cell Types**	**Species**	**Functions**	**References**
FOSLI	Binds to migration enhancers during trophoblast invasion. Dimerizes with JUN to form AP-1 complex.	Extravillous trophoblast cells	Mouse and human	Involved in trophoblast differentiation and migration.	[1,13,56,57]
TFAP2C (human) or Tcfap2c (mouse)	Interacts with TFAP2C to recruit TFAP2C to double- or single-occupancy sites in differentiation genes in TS cells.	TS cells, Invasive trophoblast cells	Mouse and human	Involved in trophectoderm lineage determination, placental development, and trophoblast differentiation.	[13,15,44,45,46,47]
**Trophoblast-Specific TFs**	**SE Association**	**Trophoblast/Placental Sub-Cell Types**	**Species**	**Functions**	**References**
ERG	Regulates CDH5, ANGP2, DLL4, CLDN5, and VW for vascular development and angiogenesis.	Endothelial cells	Mouse and human	Involved in vascular development and angiogenesis.	[1,21,56,66,69,70,71]
GATA-2 and GATA-3	Regulates the expression of hormone PLF along with GATA 3.	Murine trophoblast giant cells, Cytotrophoblast progenitors	Mouse and human	Involved in endothelial cell migration, neovascularization, and angiogenesis.	[51,73,74,75,76,77]
ETS2	Regulates TS cell renewal by regulating CDX2 and trophoblast invasion via MMP-3, MMP-9, and MMP-13.	TS cells	Mouse and human	Regulates trophoblast differentiation and invasion.	[13,53]

## 5. Role of Super-Enhancers in Placental Diseases

### 5.1. Super-Enhancers in Disease Pathogenesis

SEs have been implicated in the pathogenesis of various diseases, as they can have altered functions due to epigenetic alterations, changes in enhancer sequences, or chromosomal rearrangements [78]. Enhancer-mediated mechanisms have now been increasingly linked to cancer and other rare diseases, including congenital disorders and neurodegenerative diseases [78]. If an association of an SE cluster with a specific disease is identified, then the target genes they regulate can be used as disease biomarkers and to develop drug targets. It is known that disease-associated polymorphisms are within non-coding DNA regions and that the most quantitative trait loci (QTL) are expression QTLs (eQTLs). Thus, it is unsurprising that SEs are associated with disease development [20,50,56], and that disease-associated variations are enriched in SEs [26]. In their ChIP-Seq analysis of various human tissues, Hnisz et al. found SNP-enriched SEs in human brain tissues at loci associated with Alzheimer’s disease. Furthermore, genetic variants associated with rheumatoid arthritis were found within lymphoid cell SEs [26].

SEs have also been implicated in states of pathological proliferation, such as cancer. ChIP-Seq data revealed that cancer cells could accumulate SEs as proto-oncogenes mutate into oncogenes, resulting in the unregulated expression of SE-associated genes and subsequent tumorigenesis [26]. One example is the oncogene MYC proto-oncogene, BHLH transcription factor (MYC), a cell growth and apoptosis regulator. ChIP-Seq enriched MYC in nearby SEs in cancer cells, but not in their healthy counterparts [20,26,79]. A separate study of multiple myeloma found that MYC-associated SEs were occupied by remarkably high amounts of the coactivator bromodomain containing 4 (BRD4), a protein involved in the recruitment of elongation factors and binding of Mediator, as well as acetylated histones [80,81]. Furthermore, the inhibition of BRD4 was associated with selective disruption of SEs, thereby implicating the coactivator as a SE-associated driver of tumorigenesis [20]. BRD4 binding to SEs has been conserved across placental mammals, and its inhibition causes reduced pluripotency and activation of SOX2 SEs in porcine stem cells [23]

### 5.2. Super-Enhancers in Placental Diseases

**Figure 3:**
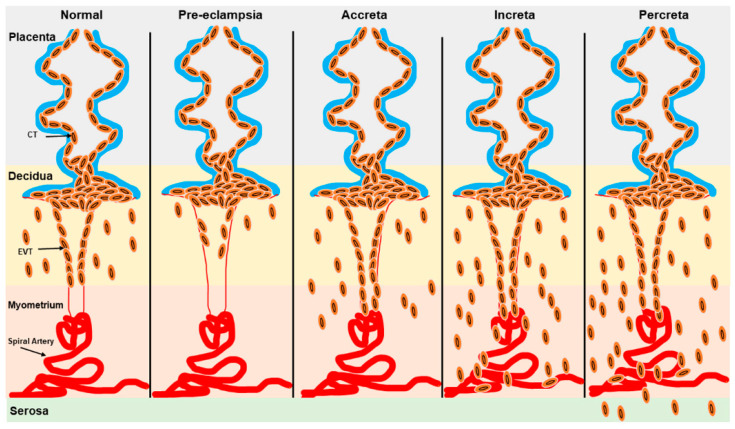
Schematic representation of trophoblast invasion in normal and pathogenic states. Trophoblast invasion for spiral artery remodeling is severely restricted in the decidua in PE while excessive trophoblast invasion in the myometrium and serosa is observed in placenta accreta, placenta increta, and placenta percreta. EVT: Extravillous trophoblast; CT: cytotrophoblast.

Defective trophoblast differentiation and invasion of the decidual spiral arteries during pregnancy are central to many pregnancy complications, including PE, which is associated with significant maternal and fetal morbidity and mortality worldwide [82,83]. While the exact etiology and pathophysiology of PE remain incompletely understood, a key factor is the failure of extravillous trophoblast cells to properly remodel the spiral arteries [84]. This failure reduces blood flow to the intervillous space, contributing to fetal growth restriction (FGR) and maternal symptoms of PE [85,86]. (Figure 3). Conversely, excessive trophoblast invasion during pregnancy involves placental diseases such as placenta accreta spectrum (PAS), which includes placenta accreta, placenta increta, placenta percreta, and placenta previa (Figure 3). In PAS, trophoblast invasion can extend into various layers of the myometrium and even the uterine serosa, resulting in life-threatening hemorrhage that often necessitates interventions like cesarean sections or hysterectomy [85,86,87,88] (Figure 3). Placenta previa (PP) specifically involves the abnormal implantation of the placenta near the cervix, obstructing the internal os and leading to inflammation, which in turn causes maternal–fetal morbidity and mortality [85,86,89,90]. Preterm births with various etiologies are associated with different neonatal complications. For instance, infants born after spontaneous labor are at a higher risk of intraventricular hemorrhage. In contrast, those delivered preterm due to iatrogenic factors face an increased risk of necrotizing enterocolitis, coagulopathy, and pathologic hypoglycemia [91].

Studies on the pathogenesis of PE involving human term placentas, in vitro cultures, and placental perfusion studies by Doppler, fail to provide mechanistic clues on the onset of this disease [92]. To overcome these difficulties, mouse and rat models of PE have been created by either inducing inflammation, immune system imbalance, angiogenic imbalances via sflt1, genetic manipulation of the renin–angiotensin–aldosterone system (RAAS), or placental ischemia [93,94]. However, none of these models have been able to completely recapitulate the physiological effects observed in humans, and thus provide limited information on the pathogenesis of this disease. Most of these animal models do not replicate the initial events involved in PE, especially where SEs will be increasingly implicated [94].

The lack of studies on the functional significance of SE networks in human placentation has hampered the understanding of the disease pathologies of PE, PAS, and placenta previa. Nonetheless, only a few TFs have been implicated in the pathogenesis of PE [1]. For example, the SE-upregulated gene HTRA1 stabilizes misfolded proteins while GATA3 is involved in placental imprinting in PE [95,96]. Another SE-regulated gene, syndecan 4 (SDC4), is also up-regulated in PE, possibly due to the presence of an inflammatory environment [97].

Enhanceosome formation due to combinatorial binding and collaborative actions of multiple TFs on SEs activates several target genes due to a transcriptional synergy in human TS cells. On the other hand, repression of target genes may result when these TFs function singly or are associated with fewer TFs [98]. In regions where the five SE-enriched TFs, FOS, TEAD4, TFAP2C, GATA2, and MAF BZIP transcription factor K (MAFK), co-bind in TS cells, the expression of target genes is significantly higher than in regions where there is only the binding of single TF or few TFs. The genes associated with the pathogenesis of PE mainly belong to the group of target genes activated by the combined binding of all five TFs [98]. Thus, deregulations in co-binding and actions of multiple TFs to SE regions appear essential in developing PE and other diseases.

**Figure 4:**
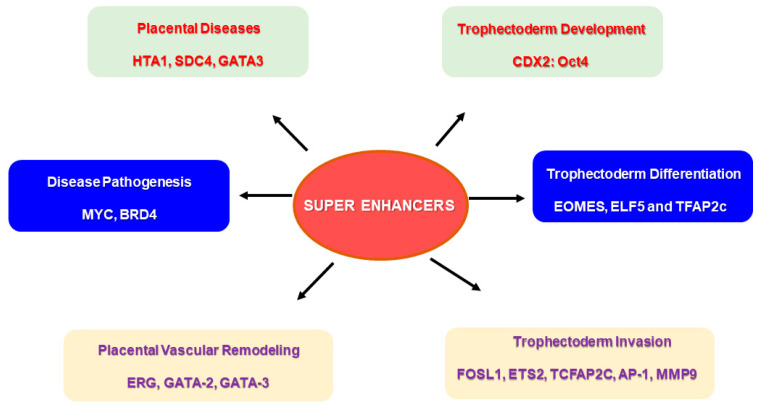
Super enhancers (SEs) and their putative role in placental physiology. Specific super enhancer-driven networks may have a significant role to play in events required for successful placentation. SE-driven networks can be associated with trophoblast development and differentiation, and trophoblast invasion of spiral arteries, which are important for vascular remodeling during pregnancy. Several SE-driven events may also be responsible for disease pathogenesis, specifically in placental diseases.

The combinatorial interplay of multiple TFs and their binding to SEs may be required to stimulate cell type-specific gene expression during placental development [98] (Figure 4). TS cells and cytotrophoblast cells exhibited a higher expression of target genes when all five TFs were bound to the SE regions compared to syncytiotrophoblast and extravillous trophoblast cells [98]. It is worthwhile to assess the mechanism by which these TFs bind to SE regions and create functional networks by activating genes during trophoblast development and invasion. Derangements in such mechanisms may be significant in understanding the pathology of placental diseases and aiding in developing treatment strategies. Placental dysfunctions contributing to pregnancy complications, such as preeclampsia and preterm birth, can lead to adverse neonatal outcomes. Investigating the role of SEs in regulating the signaling pathways associated with preterm birth may provide insights for reducing its incidence and the related neonatal complications [99].

## 6. Conclusions

Although the precise roles of SEs in placental development and pregnancy complications related to defects in placental development are not yet fully understood, emerging evidence suggests that SEs play a significant role in driving the expression of several genes crucial for placental development (Table 1). We recognize that there is still much to be discovered, as SEs represent a relatively new and rapidly evolving area within the fields of epigenetics and transcriptional regulation. The identification of SE-associated TFs is an essential first step toward unraveling the full spectrum of SEs’ functions. Given that SEs rarely act in isolation and commonly share TFs and cofactors, there is much more to explore. Looking ahead, we call for further research into the role of SEs within enhancer landscapes and regulatory networks. The continued characterization of SEs and their associated interactions will deepen our understanding of abnormal placentation and may ultimately uncover novel therapeutic targets for placental diseases. Additionally, investigating SE-associated alterations in chromatin dynamics and the long-range chromatin interactions that drive trophoblast differentiation could be mapped using 5C- and Hi-C-based approaches. With the rapid development of bioinformatics tools like HOMER (http://homer.ucsd.edu/) and ROSE (Rank Order of Super Enhancers), mapping these elusive DNA elements that direct the placental developmental program is feasible.

## Data Availability

Not applicable.

## Abbreviations

The following abbreviations are used in this manuscript:

SE	Super-enhancers
TS cells/TSC	Trophoblast stem cells
ICM	Inner cell mass
TFs	Transcription factors
TGCs	Trophoblast giant cells
CDX2	Caudal-type homeobox 2
EOMES	Eomesodermin
TCFAP2C	Transcription factor AP-2 gamma
GATA3	GATA-binding protein 3
ETS2	ETS proto-oncogene 2, transcription factor
ELF5	E74-like ETS transcription factor 5
ESSRB	Estrogen-related receptor beta
SOX2	SRY-box transcription factor 2
ID1	Inhibitor of DNA binding 1
TEAD4	TEA domain transcription factor 4
SATB1	SATB homeobox 1
TP63	Tumor protein P63
OCT4	POU class 5 homeobox 1
NANOG	SOX2, and Nanog homeobox
PIM1	Pim-1 proto-oncogene, serine/threonine kinase
FGFR2	Fibroblast growth factor receptor 2
CBP	Creb-binding protein
SMC	Cohesion structural maintenance of chromosomes
HUVECs	human umbilical vein endothelial cells
CDH5	Cadherin 5
ANGP2	Angiopoietin
DLL4	Delta like canonical notch ligand 4
CLDN5	Claudin 5
VWR	Von Willebrand factor (VWF)
FOSLI	Fos proto-oncogene, AP-1 transcription factor subunit 1
JUM	Jun proto-oncogene
MMP9	Matrix metalloproteinase 9
MYC	MYC proto-oncogene, BHLH transcription factor
BRD4	Bromodomain containing 4
MAFK	MAF BZIP transcription factor K
